# Exploring the lived experience of economic insecurity and health among people accessing charity-run food provision services in Bristol, UK

**DOI:** 10.1017/S002193202500001X

**Published:** 2025-01

**Authors:** Geneviève Stone, Angeliki Papadaki

**Affiliations:** 1 Centre for Exercise, Nutrition, and Health Sciences, School for Policy Studies, University of Bristol, Bristol, UK; 2 Department of Anthropology & Archaeology, University of Bristol, Bristol, UK

**Keywords:** food insecurity, UK, qualitative research

## Abstract

The UK has experienced alarming increases in the number of individuals living with food insecurity as a result of the rise in the cost of living. The mechanisms linking household economic insecurity to food insecurity, and perceived health outcomes, are not well understood. The aim of this study was to explore how individuals with lived experience of food insecurity are coping with the rise in the cost of living, the trade-offs they might be making between food and other household expenses, and how these might impact eating behaviours and health outcomes. Using a qualitative inductive approach rooted in hermeneutic phenomenology, nine semi-structured interviews were conducted among individuals using charity-run food provision services in Bristol, UK. Narrative accounts from these interviews were analysed thematically. Almost all participants were recipients of benefits at the time of interviews and were living under high levels of economic insecurity. The rise in the cost of living forced complex budget management strategies, including relying on donated food and shoplifting. It also influenced eating behaviours through altered cooking strategies to save energy, substituting food for cheaper, less-nutritious, alternatives, and rationing meals. Food insecurity was experienced as a form of psychosocial violence, engendering high levels of stress, particularly for individuals with diet-related chronic diseases. There is therefore an urgent need for policies that tackle structural causes of overall household economic insecurity, and improve economic access to adequate nutritious foods, to prevent further entrenching social inequalities.

## Introduction

Food security is defined as ‘*a situation where all people, at all times, have physical and economic access to sufficient, safe, and nutritious food to meet their dietary needs and food preferences for an active and healthy life*’ (FAO, 1996). The absence of food security is therefore characterized by uncertainty about whether one will be able to access food of adequate quality and quantity in ways that are socially acceptable (Nord, 2014). In the United Kingdom (UK), an estimated 9.3 million adults, or 17.7% of UK households, experience food insecurity (The Food Foundation, 2023). Since the early 2010s, scholars in the UK have been alerting of the link between austerity measures and food insecurity reaching epidemic proportions (Taylor-Robinson *et al.*, 2013; Garthwaite, Collins and Bambra, 2015). The COVID-19 pandemic, combined with the recent unprecedented rise in the cost of living in the UK, has exacerbated food insecurity further. This is particularly true for poorest households, who, on average, spend a larger proportion of their income on essential commodities, such as energy and food (Limb, 2022). Not only is food insecurity a matter of social concern but it is also a public health issue given that it has been widely associated with adverse health outcomes, including undernutrition, obesity, and poor mental health (Swinburn *et al.*, 2011; Jones, 2017).

In the UK, cross-sectional and longitudinal studies prior to the cost of living crisis suggest that food insecurity is strongly associated with socio-economic status, education level, and receipt of benefits (Tingay *et al.*, 2003; Pilgrim *et al.*, 2012; Power *et al.*, 2018; Loopstra, Reeves and Tarasuk, 2019). Most of the UK research on food insecurity has also focused on people accessing formal food banks, where a referral is required to receive assistance (Garthwaite, Collins and Bambra, 2015). There is a paucity of evidence on the network of other support systems that exist in the UK, which do not require proven need to be accessed. People accessing food support via these other means remain invisible to policymakers, as they are not counted in official figures of food bank users, and their experiences are not included in discussions around social protection mechanisms and food poverty. Moreover, it remains unknown how individuals who find themselves in this position negotiate cost allocation between other household expenditures and food, nor how such trade-offs may link to perceived health outcomes. Understanding how individuals navigate the experience of economic insecurity as it relates to food choices and health (both mental and physical), especially when they are seeking support outside of formal networks, is crucial to devising better policies that promote health and well-being.

Currently, there is no international consensus on how food insecurity should be measured (Lambie-Mumford and Dowler, 2015). Historically, food insecurity has been measured through quantitative methods by economists, where the focus was on supply and demand at the national level and on energetic sufficiency for individuals (Habicht *et al.*, 2004). In the last two decades, the use of ‘experience-based scales’, which measure individual perceptions of one’s food situation, has become widely adopted (Piperata and Dufour, 2021). This approach represents an advance in capturing different levels of food insecurity and, to some extent, variations in nutritional quality. However, these are blind to most coping mechanisms that occur within households, such as decision-making around acquisition, food preparation, and allocation (Piperata and Dufour, 2021). For example, these scales do not capture variability in eating times, which has been highlighted as potentially a key driver of the relationship between food insecurity and obesity (Nettle and Bateson, 2019). These topics therefore remain understudied, yet are key to understand the mechanisms linking food insecurity and health outcomes. There has also been no methodological consistency in the tracking of food insecurity prevalence in the UK, which makes it challenging to monitor rates at the national level (Pool and Dooris, 2022). Charities in the UK have advocated for the adoption of the FAO’s Food Insecurity Experience Based Scale (FIES) to harmonize these data and generate internationally comparable figures (Food Foundation, Sustain, and Oxford University, 2016).

To address the aforementioned gaps in the evidence base, the current study aimed to explore the perceptions of people accessing charity-run food provision services in Bristol, UK, around the link between economic insecurity and different dimensions of food insecurity (stability, access, availability, and utilization), the mechanisms used to cope with food insecurity and how these shape the utilization of food, and the link between economic and food insecurity and physical and mental health.

## Methods

The Consolidated criteria for Reporting Qualitative research (Core-Q) checklist was used in the reporting of this article (see supplementary material 1) (Tong, Sainsbury and Craig, 2007).

### Study design

The study utilized a hermeneutic phenomenology approach, which aims to understand the lived experience of a particular group of people experiencing the same phenomenon through observation and one-to-one interviews (Flood, 2010). Embodiment is central to phenomenology, as it is from the body that knowledge and perceptions arise, and therefore inscribe the cultural onto the biological (Taylor, 1989). In this project, the aim was to understand how economic insecurity is perceived and embodied through food choices and eating behaviour among people experiencing the ‘phenomenon’ of food insecurity.

### The field site

The study took place over the period of June and July 2022 in the Easton and Barton Hill localities of the City of Bristol (UK). These localities are among the 10% most deprived in the country and rank particularly low in terms of income and employment (Ministry of Housing, Communities & Local Government, 2019). On average, 32% of people report below-average mental well-being in the most deprived areas of Bristol and an estimated 11% of households in such areas experience food insecurity (Bristol City Council, 2022). A further 5% of people living in the most deprived localities of Bristol reportedly use food banks or charities to support themselves (Bristol City Council, 2022). However, in terms of access to local food vendors, markets, and bigger supermarket retailers, both neighbourhoods are well serviced.

The fieldwork was carried out in partnership with FoodCycle, a volunteer-run charity that provides a free home-cooked meal on a weekly basis, which anyone can consume at their two different sites. Individuals do not need to sign up to this service, nor prove need to access the free meal, unlike other organizations, such as food banks. FoodCycle also offers ‘groceries’ that individuals can take home, which mostly consists of a free-for-all table with a variety of fruits, vegetables, and occasionally breads that are just out of date, or about to spoil, but can still be safely consumed. Recipients of the service can help themselves to these groceries before their meal and take them home in bags that they bring themselves.

### Participant recruitment

Participants were recruited through FoodCycle during the bi-weekly meals in Barton Hill and Easton. An email was sent to the FoodCycle regional manager explaining the purpose of the study with a request to approach individuals attending their meal services (whilst they were arriving at the dining hall sites) to take part in an interview. An opportunistic sampling approach was taken, as it was most appropriate to the research setting (Bernard, 2017). To be eligible, participants had to be over 18 years of age, use the services provided by FoodCycle, and be current residents of a City of Bristol locality. Participation was voluntary. All participants were provided with written information about the study before giving consent to take part in the interviews. Participants were offered a 15GBP supermarket voucher to compensate for their time spent during the interview. No participants refused to participate when they were approached and invited to take part. Participants were recruited until data saturation was reached.

### Data collection

Semi-structured interviews (lasting 20–55 minutes) were conducted and audio recorded in the FoodCycle dining hall premises in a confidential space that was still within view of volunteers for safeguarding purposes. No notes were taken during the interviews. The questioning guide was developed based on the interview guide utilized by Puddephatt *et al* (Puddephatt *et al.*, 2020), extended to include more questions that explore wider contexts of household economic insecurity, stress, and perceived health. A hermeneutic phenomenological interviewing approach was used to develop and adapt the guide (Manen, 2016; Lauterbach, 2018), which followed a conversational format, whilst using targeted questions to explore key themes of the lived experience of economic and food insecurity (see supplementary material 2 for targeted questions) (Manen, 2016). Interviews were conducted in English by the first author, a trained qualitative researcher who holds an MAnth in Medical Anthropology, and were transcribed verbatim and anonymized. Data transcripts were not returned to participants for comments due to the transience of people who use FoodCycle services.

Survey-type questions on background demographic characteristics, as well as the Food Insecurity Experience Scale (FIES) and the Perceived Stress Scale (PSS), were administered to participants. These scales were used in order to be pilot-tested in view of a future project and critically reflect on current approaches to measure food insecurity and stress (Lauterbach, 2018; Piperata and Dufour, 2021). As such, the data from these scales will only be presented to provide context to the qualitative data generated, but not analysed further for the purpose of this paper.

### Analysis

The data analysis and interpretation were executed in three main steps, guided by a hermeneutic stance, which is inherently inductive. The first step involved reading each of the anonymized transcripts in their entirety and making notes manually about elements that stand out in the text (Crist and Tanner, 2003). Based on this step, an initial codebook with emergent themes was developed using NVivo (version 12.0, QSR, Southport, UK, 2018) for each transcript. A list of codes for all interviews was compiled upon this first reading and used as a basis for the second step. In the second step, a circular approach was adopted, known as the ‘hermeneutic circle’ (Crist and Tanner, 2003). This step entailed refining the identified themes by constantly returning to specific passages of the transcript, as well as comparing themes across different transcripts (Crist and Tanner, 2003). The third step involved discerning an ‘end’ to the circular approach and consolidating themes into a contextualized and credible account of what was uncovered during fieldwork (Crist and Tanner, 2003). Selected quotes from individuals are presented with the participant code (ranging from P01 to P09), gender (indicated as male/female, M/F), and food insecurity status according to the FIES score (secure, mild FI, moderate FI, or severe FI). Data coding and analysis were conducted by the lead author.

### Author positionality statement

Whilst neither of the authors have directly experienced food insecurity, the lead author had volunteered regularly with FoodCycle and other community organizations prior to this study. As such, the lead author was already known to participants when she began interviews, and the aims of the research were clearly stated to both the gatekeeper organization and participants. The second author was not directly involved in data collection or analysis, but rather provided input in the project design and review of the manuscript. Both authors disclose a prior interest in food security and approach research on this topic from a rights-based perspective.

### Ethical considerations

Ethical approval was obtained by the School for Policy Studies Research Ethics Committee (approval code 11001).

## Results

### Study sample characteristics

**Figure 1.**
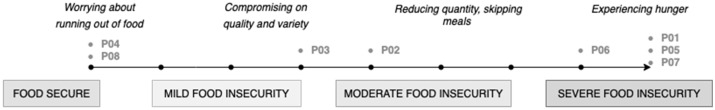
Food Insecurity Experience-based Scale (FIES) with relative participant positions.

Participant characteristics are demonstrated in Table 1. In total, nine participants (five women and four men) took part. Only one participant was retired and only one did not receive any kind of benefit support from the government (but indicated relying on her full-time employed partner). All participants indicated being regular users of the FoodCycle community meals, and all reported that they were not regular users of formal food banks, as they did not have permission to use these based on their income or benefits status. Based on the FIES scores, four participants were classed as severely food insecure, one as moderately food insecure, one as mildly food secure, and two as food secure (Figure 1).

**Table 1.** Socio-demographic characteristics of participants

Participant Code	Gender	Employment	Receives benefits	FIES score	FIES Classification
P01	Female	Unemployed	No	8	Severe FI
P02	Male	Part-time employed	Yes	4	Moderate FI
P03	Female	Unemployed	Yes	3	Mild FI
P04	Female	Part-time employed	Yes	0	Food secure
P05	Male	Unemployed	Yes	8	Severe FI
P06	Male	Unemployed	Yes (Universal Credit)	7	Severe FI
P07	Male	Unemployed	Yes	8	Severe FI
P08	Female	Retired	Yes (pension)	0	Food secure
P09	Female	Part-time employed	Yes	6	Moderate FI

FI, food insecurity; P, participant.

**Figure 2.**
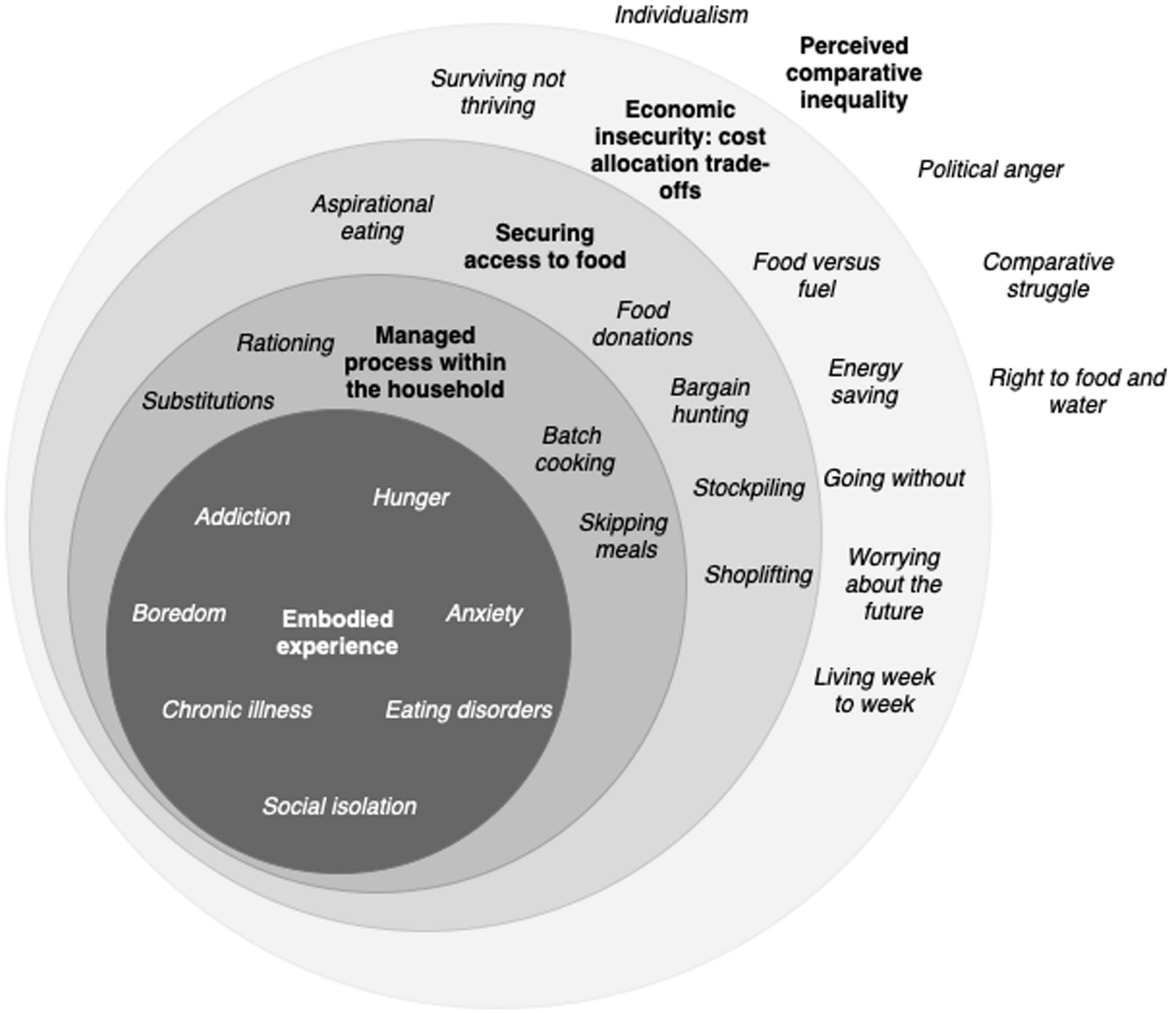
Themes and sub-themes that emerged from the phenomenological analysis of interview data. Core themes are highlighted in bold, and sub-themes are indicated in italics. These refer to the range of concepts and behaviours that emerged in relation to core themes.

The five primary themes and twenty-six sub-themes generated from the qualitative interviews are outlined in Figure 2. These show that the embodied experience is central to understanding how participants perceived insecurity to affect them, and how they proceeded with decision-making around food and household finances. Starting on the outer circle, findings suggest that perceived inequality and social position shape participants’ experiences of economic insecurity. Economic insecurity encompasses the decision-making process that participants face in allocating resources to other essential household expenses and food, as well as the increased pressure that they are experiencing due to the rise in the cost of living. The next ring exists within the wider setting of financial insecurity, but pertains specifically to strategies linked to securing food. These include a range of behaviours to save costs, whilst accessing food that is within economic means and physical availability. Within this lies the management of food at the household level, which encompasses eating patterns to maximize the use of food that was acquired. This inevitably was suggested to shape the embodied experience of individuals, as participants described physical sensations linked to hunger, chronic illness, and mental manifestations of insecurity, such as anxiety and stress. Figure 2 can be interpreted as the outer layers overlapping to shape the central circles (the lived experience), but equally the information flows from inward perception to outward behaviour, as food is one of the most central elements of the human experience.

### Embodied experience

A recurrent word that emerged from the interviews was that of ‘worry’, used by participants in reference to both food, and their economic situation. Specifically, five participants highlighted the mental toll of economic food insecurity on them as being a major difficulty in their lives. Moreover, four participants mentioned that their body weight frequently changes because they eat irregularly, and two mentioned that they felt they were overweight and had to lose weight. Most participants connected both their bodily experience and physical health, with the process of managing food insecurity, as well as their mental health. For instance, when thinking about the impact that worrying about food has on her health, one participant described it as:

*‘I can work myself into a major panic attack and anxiety. Because I know that I am Type II Diabetic now, I’ve got to eat on a regular basis and that is hard’. (P01, F, Severe FI)* Another participant described the physical sensation of being hungry as *‘You know when you can’t sleep and your stomach is gargling and bubbling and growling at you, that’s when I know okay, I should eat now’,* and the effect it has on his mental health as: *‘But the thing is with my depression anyway it’s easier than you think to not eat. You are still hungry-ish, but it takes too much energy. (…) I mean it does affect me, it affects my sleep, it affects who I am’. (P06, M, Severe FI)*

Three participants described having chronic health conditions linked to diet that they needed to manage through their food intake; all three were classed as severely food insecure on the FIES scale. Managing chronic illness was described as particularly difficult to cope with because of the lack of choice that comes with food insecurity. These participants mentioned multiple times that the food that they were receiving for free from other food donation charities was harmful to their health. One participant summed up the frustration with this situation as:

*‘Well no it doesn’t sustain you. I’d look at it and I think to myself: death by biscuits. It’s like, it’s depressing. Because it’s like, Okay, I’ve got food. But it’s just a thousand biscuits. Yeah. It’s like… (laughs in exasperation) yeah, because I had a heart attack. (…) (P06, M, Severe FI)’*

Two participants, both classed as food secure according to the FIES, mentioned that they struggled with disordered eating. The meals provided at FoodCycle were mentioned as a key part of overcoming disordered eating by being in the community. One participant mentioned that she struggled with cycling between over-eating and feeling guilty: *‘I feel guilty because I eat more than I need to. I’ve got about four diet books on the go at the moment. Sort of sensible eating type things’. (P08, F, Food Secure).* This participant also explained that what she feels are unhealthy eating habits are triggered by loneliness and boredom:

*‘Yeah but by the time you are my age, you eat from boredom because there’s nothing on the television and you can’t go out dancing like you used to. I can’t stand the television and I’m tired and fed up with myself. That’s when I sort of attack the ice cream’. (P08, F, Food Secure).*

Addiction to substances was also a key theme that came up as being interlinked with the embodied experience of food insecurity. Two participants mentioned having previously been drug addicts or were current users. Drug addiction seemed to dominate the lived experience of these interviewees, as worries about food were secondary to that of obtaining enough substance. Indeed, the participant who was a current drug user explained that obtaining food was not her main concern, rather her major source of stress was linked to needing income to spend on drugs:

*‘I’ve never got enough money, yeah, it just affects like money comes in one hand and out the other. Like say for example, Tuesday morning I had 450 pounds, Wednesday morning I didn’t have a penny. And I’ve got nothing to show for it’. (P03, F, Mild FI)*

### Managed the process of food insecurity within the household

Within the household, multiple strategies to maximize the use of food that was obtained were described. One of the main strategies was that of batch cooking and storing food in the freezer to avoid food waste and cope with irregular amounts of food. Two participants mentioned vegetarianism as a way of keeping food costs lower, and three mentioned it as being aspirational. This included substitutions of animal-sourced foods in meals, as well as strategies to add more carbohydrate-rich foods to meals, which were identified as being less expensive. One participant described doing this for herself and her partner, but not her child:

*‘Yeah, I mean, well I’m going more to like plant based just because meat is really expensive at the moment. And it’s just like, if I can like use mushrooms or something, so substitute. (…) So I just stick to more veg and lentils. Like the cheap stuff, or what I class as cheap stuff. (…) I think to make myself fuller I would just add like, chickpeas or something, or add carbs like potatoes or something to lengthen it out’. (P09, F, Moderate FI)*

Another strategy that was mentioned was that of eating the same meal repeatedly or drastically reducing the variety of foods consumed:

*‘Yeah, sometimes you get worried about it. And sometimes all you’ve got is beans on toast cause you know you’ve got to make it last’. (P02, M, Moderate FI).*

Furthermore, two participants described skipping meals entirely, or eating smaller portions, to ensure that the available food lasted longer: ‘*Honestly, I don’t eat three meals a day. I have one less. Maybe two meals a day so it’s fine and sometimes I only have one’*. (P07, M, Severe FI)

### Securing access to food

Multiple strategies were described by participants for obtaining food within their financial means. One of the most frequently mentioned strategies was that of utilizing FoodCycle and other organizations that provide free groceries as a primary way to obtain food and complementing this with purchased items. When shopping for food, participants described strategies to cut on costs, including bargain-hunting by going to multiple different stores, and purchasing food more frequently as money becomes available:

*‘I’ll try my best to get things that are discounted, so sometimes I’ll go late at night, so like 20 mins or half an hour before the store shuts and I’ll see what they have in the reduced to clear section. I can’t remember the last time I actually did a shop where I bought everything straight from the regular shelves’. (P06, M, Severe FI)*

Three participants mentioned stockpiling as a behaviour to secure access to food and associated this with feeling less worried about the rise in the cost of living. These were all women, and none of them were experiencing food insecurity. Two of these women mentioned that this was triggered by the COVID-19 lockdowns.

Finally, one person reported resorting to an unlawful way of obtaining food, which included paying someone to shoplift for them. This participant reported that this is a common behaviour to obtain food among other people that they knew. When asked if he was stressed about obtaining food in this way, he emphatically responded: ‘*Yeah, I mean of course, and it’s not just that, they know I’ve got money on me. They might mug me. I mean who is to stop them’*, as he reported that people offering shoplifting services are drug addicts who do not fear receiving a criminal record for illegal activity and may also take advantage of people who request their services.

### Economic insecurity: cost-allocation trade-offs

A common theme that emerged around economic insecurity was trying to reach some level of predictability with finances, rather than having to live day to day without the possibility of planning for the future. Interestingly, even when participants might have reached a more stable financial position, they described that the mindset that ‘things may suddenly become unstable’ was difficult to let go of:

*‘I’m not on the poverty impoverished line or anything like that. Up here it is a bit like when the people were at war and it was short. And when they came out and things picked up a bit they still would turn the light off, you know. And that mentality is where I’m at, it’s still…, I’m still just beginning to let it go now’. (P08, F, Food Secure)*

A recurring sub-theme was that of worrying about economic hardship in the future and specifically around the rise in the cost of living. One of the emerging concerns was that of fuel prices going up, therefore driving household expenses up. For example, one participant indicated that the insecurity he was experiencing meant he was unable to reach a position where he can plan ahead:

*‘Yes, the worry is too much. You can’t just think ahead for a month or week or two, it’s you have to think day to day, what’s going to happen tomorrow, what will increase, what’s going to be expensive’. (P07, M, Severe FI)*

This was framed as inevitably engendering trade-offs with how much of the budget can be allocated to food. Indeed, several participants explained that they had already started adjusting the allocation of their budget to different necessities or factoring this into decision-making when purchasing food:

*‘But then again, it’s the storage and the electrics as well, so you will have to think about everything when you do the shopping. You can’t just go to the shop and say, oh yeah, I’ll have X, Y, and Z, and then not have the room or, the gas or the electric to be able to keep it fresh’. (P01, F, Severe FI)*

Some participants indicated having changed their methods of cooking food in order to lower costs of energy bills. Strategies used included using the microwave or kettle instead of the oven or cooking multiple items in the oven at the same time:

*‘I avoid using the microwave for too long. (…) Why should I cook rice that’s in the packet for 15 to 20 minutes when I don’t have to eat that, I could eat couscous because it’s done with just boiling water from the kettle once. (…) I used to cook things in the oven. I used to do like tuna pasta bakes, I don’t do those anymore’. (P06, M, Severe FI)*

Another concern that was highlighted by four participants was that of petrol prices for cars, as many lived in areas without walking access to supermarkets, and prices were higher at the local shops. For example, one participant described:

*‘The milk, is usually a pound in the other shop, but in the local shop usually 1.20 or 1.30. But maybe if you use your car, you will be spending the same amount, so the car is only used for one trip or the essential’.* (P07, M, Severe FI).

### Agency within an unfair system: perceived inequality and political anger

Political anger was a theme that emerged in multiple interviews and was often expressed in relation to economic inequality. Indeed, some participants expressed the view that they felt resources were not well distributed and that food should not be something that people have to go without in a country as wealthy as the UK:

*‘I want to say maybe one short statement (…) to the government: you’ve got money to fund a war, but you haven’t got money to feed the poor? It’s not fair. (…)’ (P07, M, Severe FI).*

## Discussion

**Figure 3.**
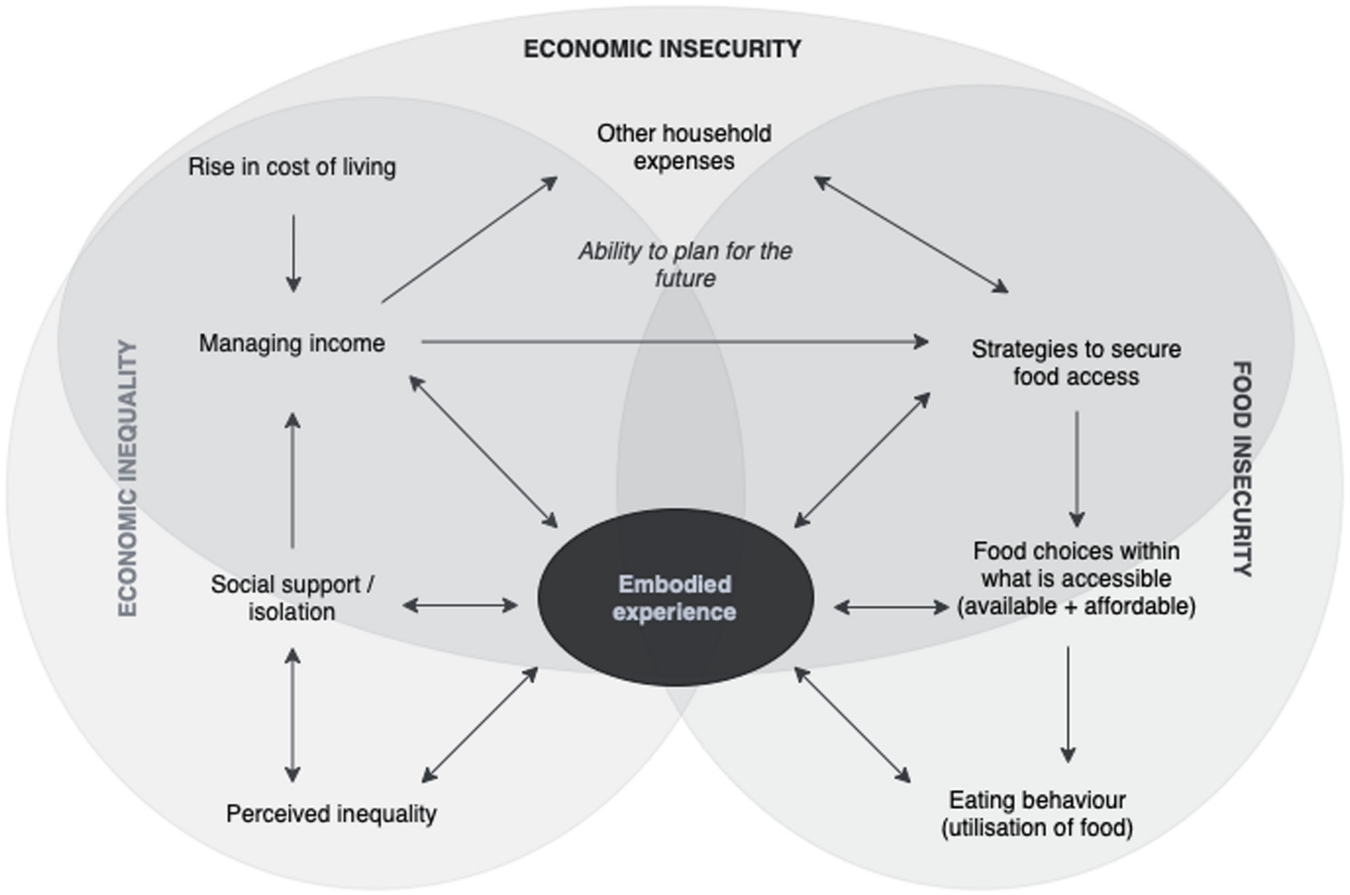
Relationship between concepts emerging from the interview data as they pertain to the three overarching concepts of perceived economic insecurity, economic inequality, and food insecurity, with the embodied experience at the centre.

The main findings from this study are visually summarized in Figure 3, which illustrates how the embodied experience is shaped by, and shapes, other processes that surround decision-making around household expenses and food. The conceptual domains of economic insecurity, economic inequality, and food insecurity overlap in their interactions, leading to what can be described as an experiential ‘system’. The rise in the cost of living was identified as a major external element putting pressure on the whole system, which is perceived to worsen economic insecurity, inequality, food insecurity, and ultimately well-being.

A key finding of this study was that economic insecurity, which has been exacerbated by the rise in the cost of living experienced since March 2022, was perceived to shape the experience of food insecurity for adults who are accessing charity food provision services. Indeed, economic insecurity was the primary source of worry for all participants, as well as the main driver of the use of FoodCycle services. This is consistent with findings from quantitative studies that link perceived economic insecurity, and receipt of benefits, to food insecurity (Rose, 1999; Power *et al.*, 2018). All, but one, participants were receiving benefits at the time of the interview, which suggests that benefits do not provide a sufficient economic safety net. There is little qualitative peer-reviewed research on this topic in the UK; however, this is consistent with evidence from grey literature commissioned by the Trussell Trust and Child Poverty Action group (Cooper, Purcell and Jackson, 2014; O’Connell *et al.*, 2019). These reports found that being on a low and unpredictable income leads to complex budget management strategies to cover basic household expenses, including food-related expenses (Cooper, Purcell and Jackson, 2014; O’Connell *et al.*, 2019). Balancing finances in such a situation could pose a significant time drain, as participants in the current study reported grocery shopping in different shops to find the cheapest options available, or grocery shopping at unusual hours. The concept of ‘time poverty’, which has been documented in low- and middle-income country contexts, also becomes applicable here, whereby the time and mental energy dedicated to money-saving strategies on basic necessities could be a structural barrier to overcoming economic precarity (Chaudhuri *et al.*, 2021).

An important finding of this study was that budget management strategies included trade-offs between ‘eating’ and ‘heating’, which is mirrored in earlier literature (Anderson, White and Finney, 2012; Lambie-Mumford and Dowler, 2015). It has been suggested that households tend to cut down on food expenditures by compromising either quality or quantity of food (or both), in order to afford energy bills (Lambie-Mumford and Snell, 2015). Research on this topic, although sparce, was published before the rise in the cost of living. By July 2022, the Consumer Prices Index had already risen by 10.1% in the previous 12 months, with the increase in food prices being the largest contributor to this change (ONS, 2022). It is therefore unsurprising that all, but two, participants felt that they were unable to reach a minimum level of ‘breathing room’ to plan ahead and were instead living week-to-week with high levels of insecurity. The two participants that did not express this worry had stockpiled non-perishable foods in response to the COVID-19 lockdowns and were still utilizing these as their main food source. This suggests that, where possible, creating a buffer for future uncertainty, such as fluctuation in food availability or energy prices, is a common budget management strategy. However, such a strategy requires reaching a minimum level of accumulated income, which might not be accessible to many individuals who solely rely on benefit support. This finding was in line with an earlier qualitative study in the UK, which found that stockpiling was one of the strategies used by parents in situations of mild, but not severe, food insecurity, whose children were home for the holidays and thus unable to access free school meals (Shinwell and Defeyter, 2021).

Participants highlighted that economic insecurity impacted on food insecurity through forcing households to resort to unconventional, or often unlawful, ways of obtaining food. Relying on gifted or free food as the main means of subsistence, and only purchasing food to supplement this, has been documented in other neoliberal high-income countries (Franklin *et al.*, 2012; Loopstra, Reeves and Tarasuk, 2019; Long *et al.*, 2020). Multiple problems with this method of acquiring food were highlighted both in this study and in the wider literature, including the poor nutritional quality of gifted food, which has been deemed to be potentially obesogenic, as well as the lack of agency in being able to choose (Tarasuk and Eakin, 2003, 2003; Middleton *et al.*, 2018). It is worth noting that in this study, nutritional quality was not perceived to be as much an issue because fruit and vegetables were the main ‘groceries’ given out by FoodCycle (in addition to the sit-in meal). Nevertheless, these problems were mentioned in relation to other free-food services which were commonly used by participants. A unique finding of the current study was that shoplifting was a strategy utilized by some in order to overcome this lack of agency and obtain foods of better nutritional quality that ‘sustain’ the body. The participant who mentioned this strategy perceived this to be common in their neighbourhood, suggesting this is something further research should explore. Shoplifting represents a more extreme means of procuring food and engenders further insecurity for the person practising it, yet it is suggested to be a tangible way for individuals to fight back on the idea that food is a commodity, rather than a human right (Whittle *et al.*, 2015; Booth *et al.*, 2018).

Narrative accounts of the lived experience of food insecurity suggest that it is a dynamic process (Garthwaite, Collins and Bambra, 2015; Beveridge *et al.*, 2019; Piperata *et al.*, 2020). A key finding from the current study was that once access to food had been secured, participants employed a range of strategies to maximize its use within the household. These strategies, including processing of food to extend shelf life, appropriate storage, and managing intake, have also been highlighted in previous qualitative studies in high-income countries (Wicks, Trevena and Quine, 2006; Booth *et al.*, 2018; Puddephatt *et al.*, 2020; Pybus, Power and Pickett, 2021). Within this set of strategies, a salient finding was the substitutions of foods, in particular meat, for cheaper and more ‘filling’ alternatives, such as starchy foods. Evidence from quantitative literature posits this substitution as a potential driver of obesity among individuals living with food insecurity (Hanson and Connor, 2014; Leung *et al.*, 2014; Morales and Berkowitz, 2016) and therefore should be explored further.

With regard to substitution patterns as a means to address food insecurity, some participants discussed vegetarianism as an aspirational form of eating. The experience of food insecurity has been highlighted in the literature as being highly tied to cultural norms around which foods are desirable or, on the contrary, associated with poverty and, thus, perceived as potentially shameful (Dufour, Lisa K. Staten, *et al.*, 1997; Dufour, L. K. Staten, *et al.*, 1997). As endorsement of plant-based diets by the UK public has increased in recent years, it is possible that this is engendering a shift in perspectives over behaviours linked to food insecurity as being less shameful (Bryant, 2019; Patel and Buckland, 2021). However, from a health perspective, such substitutions may also be linked to obesity or micronutrient deficiencies, as participants mentioned replacing animal foods with carbohydrate-rich foods, rather than foods of equal nutritional value (Seligman *et al.*, 2010; Morales and Berkowitz, 2016). Further research around how social norms link to vegetarianism within resource-constrained settings is needed in order to better understand how this emerging aspirational behaviour would impact on health.

At its core, food insecurity is one of the most embodied experiences there is, as food is what builds and sustains bodies, making it a universal basic necessity (Krieger, 2005). At the same time, being prevented from securing enough, or nutritious, food has been suggested to be a form of violence to the physical body and mind (Lindberg *et al.*, 2022). In this study, narratives around physical sensations and emotional responses to different experiences surrounding food insecurity permeated all interviews. In the ethnographic literature, as in this study, physical and mental sensations are described as interconnected, whereby physical hunger affects not only identity, but equally stress and mental health conditions, such as depression, and affects appetite by either increasing it or suppressing it (Hamelin, Beaudry and Habicht, 2002; Chilton and Booth, 2007; Piperata *et al.*, 2020). When asked how they felt about their current access to food, it is noteworthy that participants brought up struggles around eating behaviours. The range of behaviours highlighted ranged from binge eating (due to boredom and anxiety from a person who stockpiled food), to not eating for several days (due to depression and not having any food in the cupboards). These behaviours suggest that participants perceived their feelings to be shaped as a response to the material context of their daily lives (i.e. the food environment of their household). Ethnographic accounts of food insecurity among marginalized communities mirror this finding, whereby participants also describe the experience of food insecurity as a ‘hunger of the mind’ (Tarasuk and Eakin, 2003; Chilton and Booth, 2007). Indeed, the experience of food insecurity goes beyond not being able to access food due to ‘lack of resources’, as is suggested on the FIES scale (Piperata and Dufour, 2021), but rather encompasses the persistent psychosocial stress and shame of having to obtain food through non-socially acceptable means, such as food pantries.

The lived experience of food insecurity was perceived to interact strongly with illness of both the body and the mind. For participants with diet-related chronic illness, such as diabetes or heart disease, not being able to access adequate food was experienced as an additional layer of psychosocial trauma. This is consistent with other studies, where the worsening of chronic diseases as a result of food insecurity was described as engendering mental distress, manifesting as panic attacks, heightened anxiety, and depressive episodes (Puddephatt *et al.*, 2020; Rizvi *et al.*, 2022). Participants with such chronic illnesses were aware of the dietary changes that they needed to make to manage their condition, suggesting that their inability to do so is not linked to lack of knowledge or poor personal decision-making, but rather structural barriers to accessing and consuming adequate nutritious foods (Wicks, Trevena and Quine, 2006; Puddephatt *et al.*, 2020). A recent UK study concluded that there is a strong disconnect between the advice that individuals experiencing food insecurity receive from health practitioners and the reality of their daily lives, which impedes them from making such changes (Douglas, MacIver and Yuill, 2020). Indeed, participants with chronic diseases in the current study, who had been told they needed to lose weight and have a healthier diet, described striking feelings of wrestling with hunger, and having no choice but to eat highly processed foods.

The embodied experience of food insecurity cannot be discussed without consideration of the cultural and social structures that perpetuate it. During the interviews, participants frequently expressed political anger in response to the perceived social inequality under which they were living. In the literature, the embodied experience of food insecurity is largely shaped by layers of social inequities that ultimately result in health disparities across populations, thereby becoming inscribed onto the ‘biological’ (Worthman and Costello, 2009; Whittle *et al.*, 2015; Yau *et al.*, 2020). These social inequities can be attributed to the current conservative government’s neoliberal political response to the cost of living crisis; one of putting the onus on individuals to make better decisions to manage their finances and health (Department for Environment, Food, and Rural Affairs, 2022; Lang, 2022; Power, 2022).

## Strengths and limitations

A key strength of the current study is that findings centre around the lived experience of people who are not able to access formal food banks, for which referrals are needed, but rather seek other informal services to obtain free or subsidized food. This is an understudied population group, who experience persistent food insecurity yet do not qualify for food bank assistance. To the author’s knowledge, this study is also the first in the UK to explore economic insecurity and whole household economics to contextualize the lived experience of food insecurity. One possible limitation of the project is the specificity of the setting and participants who are accessing the food provision services within the same geographic area, making the findings not generalizable. Nevertheless, this project aimed to gather the diverse experiences of individuals living with food insecurity in two of the most deprived urban localities of the England until saturation was reached among these voices. Future studies should explore the constructs assessed in the current study among people who access charity-run food provision services in other areas of the UK, such as in rural areas, where food insecurity may be experienced differently.

## Conclusion

This study highlights how food insecurity is a managed process, both in terms of strategies used to acquire food and in maximizing use of food within the household, which ultimately is perceived to have consequences for both physical and mental health. For these individuals, the energy and time allocation needed to adapt and manage food insecurity, within a context of economic precarity, was perceived to be highly taxing to overall well-being. Indeed, the emphasis on experiencing food insecurity as a form of psychosocial violence to the mind and body was a recurrent theme throughout. This was particularly emphasized among individuals living with diet-related chronic illness or substance addiction. The urgency in tackling the consequences of the rise in the cost of living for individuals already struggling with food insecurity from a structural perspective is clear, as charity-run food provision services can only be a short-term fix. This highlights the need for policies that address the underlying cause of food insecurity, which in this context was income precarity due to the cost of living crisis, so that the state can guarantee the right to food of its citizens.

## Supporting information

Stone and Papadaki supplementary material 1 Stone and Papadaki supplementary material

Stone and Papadaki supplementary material 2 Stone and Papadaki supplementary material

## Data Availability

The interview guides used to collect data, and other data that support the findings from this study, are available upon request from the corresponding author.

## Supplementary material

The supplementary material for this article can be found at https://doi.org/10.1017/S002193202500001X
