# Supplementary material for: Exploring the lived experience of economic insecurity and health among people accessing charity-run food provision services in Bristol, UK
Source: J Biosoc Sci. 2025 Feb 3;57(1):1–16. doi: 10.1017/S002193202500001X (PMC13439167; doi:10.1017/S002193202500001X)
Supplement: Supplementary file 2 [file S002193202500001Xsup002.docx]

**Exploring the lived experience of economic insecurity and health among people accessing charity-run food provision services in Bristol, UK**

*Please note that this guide lists only the questions that were included in the qualitative interview section. Additional questions administered to pilot the use of the food insecurity FIES scale, and the 10-item Perceived Stress Scale (PSS) are not included in this guide. For the full data collection guide, including the initial scripts that were shared with participants regarding information on the study, contact the lead author via email.*

**Interview question guide**

Icebreaker question: Do you enjoy cooking? What is your favorite food to make or eat?

a- What are the main ways that you obtain food in your household?

*Follow-up: Where do you tend to shop? Do you use any other ways to access food (such as allotments, food provision services, family member garden)?*

b- What kinds of foods do you tend to choose for your household?

*Follow-up: What are the main factors that influence these choices? (e.g. do you tend to chose foods based on price, or do you chose foods that you know are healthy?) Can you give me any examples of the types of foods you buy regurlarly and other types of foods you only buy once in a while? Do you buy any special foods for kids?*

c- How do you feel about your household’s current access to food?

*Follow-up: Do you ever worry that food might run out before you can buy any more?*

d- If you experience stress or worries about your food supply, how do you cope with this?

*Follow-up: How does this influence your thoughts, feelings and behavior? Do you use any strategies to make food last for longer in your household?*

e- Do you feel like your access to food affects your life in other areas? For example, do you feel that it has an impact on your physical or mental health? *Follow-up: In what way (both positive and negative)?*

f- How would you describe the financial situation of your household at the moment?

*Follow-up: Relative to others in your community would you say that you are doing better or worse off? Relative to others in the country?*

g- Do you feel that your financial situation protects you from any difficulties in the future? For example, do you have any support that you can fall back on financially?

*Follow-up: What are some strategies that you use to feel more confident about your household income?*

h- In what way has the rise in cost of living affected your household economic situation?

i- Do you think you have had to take any measures to change your budgeting or expenditure in the last year, and the last month specifically? What measures are these?

*Follow-up: Do any of these measures impact on your decisions around food and your diet for yourself or your household?*

j- What would you say is the main source of stress or worry in your life? For how long has this been the case?

k- What ways do you use to help you cope with these stressors? *Probe: if any are related to food consumption*

l- If you had unlimited money, what would your food choices be like?

*Follow-up: Would these differ to your current food choices? If so, in what way? Anything you would like to be able to buy more of, or less of?*

*Ask if there’s anything else that they would like to share with that hasn’t yet been covered in the questions.*
